# Application of Deep Eutectic Solvents and Ionic Liquids in the Extraction of Catechins from Tea

**DOI:** 10.3390/molecules25143216

**Published:** 2020-07-14

**Authors:** Sylwia Bajkacz, Jakub Adamek, Anna Sobska

**Affiliations:** 1 Department of Inorganic, Analytical Chemistry and Electrochemistry, Faculty of Chemistry, Silesian University of Technology, Krzywoustego 6, 44-100 Gliwice, Poland; anna.sobska@gmail.com; 2 Biotechnology Center of Silesian University of Technology, Krzywoustego 8, 44-100 Gliwice, Poland; 3 Department of Organic and Bioorganic Chemistry and Biotechnology, Faculty of Chemistry, Silesian University of Technology, Krzywoustego 4, 44-100 Gliwice, Poland; jakub.adamek@polsl.pl

**Keywords:** green extraction, deep eutectic solvents, ionic liquids, catechins, tea leaves

## Abstract

This work aimed to comprehensively evaluate the potential and effectiveness of deep eutectic solvents (DESs) in the extraction of seven catechins from various tea samples. Different combinations of DES were used, consisting of Girard’s reagent T (GrT) in various mixing ratios with organic acids and choline chloride. The yields of the DES extractions were compared with those from ionic liquids and conventional solvent. DES contained malic acid, as the hydrogen bond donors showed a good solubility of catechins with different polarities. In the second part of the study, a solid-phase extraction (SPE) method was applied to the extraction of catechins from tea infusions. The method was applied to the determination of selected catechins in tea leaves and tea infusions. Furthermore, we demonstrated that the proposed procedure works well in the simultaneous monitoring of these polyphenols, which makes it a useful tool in the quality control of tea.

## 1. Introduction

Tea is the most popular beverage consumed in the world, and contains significant levels of polyphenols, especially catechins. Based on species, season, horticultural conditions, and degree of oxidation during the manufacturing process in tea samples, different catechins can be present [1,2].

Recent findings indicate that flavonoids, in particular catechins, possess rather potent antioxidant properties, which may result in numerous health benefits. Based on the present results, it can be said that regular consumption of green tea can reduce the incidence of cancer, including colon, pancreatic, and stomach cancers, as well as other diseases [3,4]. Thus, it is recommended to eat products that contain large amounts of catechins, to which undoubtedly include tea [5]. The extraction and isolation of catechins from tea leaves have been achieved using numerous methods [6,7] such as solid–liquid extraction (SLE) with different solvents (e.g., methanol, acetone, ethanol, acetonitrile, water, acetate, *n*-butanol and *n*-hexane) [8,9,10,11], dispersive liquid–liquid microextraction (DLLME) [12] microwave assisted extraction (MAE) [13], supercritical fluid extraction (SFE) [14,15], and ultrahigh-pressure extraction (UPE) [16].

Owing to catechins’ potential for improving human health and extending the shelf-life of food products, an efficient and safe extraction system, preferably using green solvents, is necessary. This will promote accurate quantification of catechins in tea and tea products, as well as creating an efficient step for the isolation of individual catechins [17].

In recent years, ionic liquids (ILs) and deep eutectic mixtures have shown great potential in extraction processes relevant to several scientific and technological activities. ILs possesses many advantageous properties, such as chemical and thermal stability, nonflammability, high ionic conductivity, and a wide electrochemical potential window. These unique properties have triggered extensive studies into ILs as solvents or co-catalysts in various reactions including organic catalysis, inorganic synthesis, biocatalysis, and polymerization [18]. Furthermore, they have also been successfully applied in various areas of analytical chemistry, especially in a separation of analytes. To the best of our knowledge, the application of IL or catechin extraction from tea samples has yet to be examined [19,20].

Deep eutectic solvents (DESs) are a group of emerging solvents with excellent properties including negligible volatility at room temperature, non-inflammability, and high viscosity, as well as being environmentally benign [21]. The advantage of DESs is also the possibility of the extraction of a wide range of non-polar and polar compounds [22]. The increasing range of available DESs is due to the majority being easy to prepare, inexpensive, and biodegradable, which has provoked their application in wide and in diverse fields of science [23]. Recently, some procedures of catechin extraction from tea samples have been described using DESs [24,25,26,27,28,29].

In this study, we aim to (1) evaluate the ability of new DESs and ILs to extract catechins from tea, (2) optimize a supported solid–liquid extraction (SLE) parameters using DES as the solvent, (3) develop a fast and sensitive UHPLC-UV method for comprehensive analysis of seven catechins (catechin (C), epicatechin (EC), epigallocatechin (EGC), epicatechin gallate (ECG), epigallocatechin gallate (EGCG), gallocatechin (GC), gallocatechin gallate (GCG)) in tea samples, and (4) determine the catechins in tea leaves using the DES–SLE–UHPLC–UV method and infusion tea using the solid-phase extraction (SPE)–UHPLC–UV method.

## 2. Results and Discussion

### 2.1. Chromatographic Separation

In the first part of the study, a rapid UHPLC–UV method for the determination of catechins in tea matrices was developed. Fast separation is crucial for analyzing vast numbers of samples in order to save both solvent and time. For this purpose, a mixture of seven catechin standards was analyzed in the reversed-phase system, using three different columns (Poroshell 120 SB-C18 (100 × 4.6 mm, 2.7 μm), Poroshell XDB-C18 (50 × 2.1 mm, 1.8 μm), Poroshell 120 EC-C18 (50 × 3.0 mm, 1.8 μm)). For each column, different gradient profiles were tested, with the aim of obtaining the standards retention times and peak widths. Among all columns used, the best average resolution in the shortest time of analysis was obtained using Poroshell 120 SB-C18 (100 × 4.6 mm, 2.7 μm) column. The Poroshell 120 SB-C18 column, due to the unique, superficially porous particles and 2.7 μm particle size providing a shorter diffusion path for solutes, minimized peak broadening at high flow rates and had a high permeability. Moreover, this column enabled robust symmetrical peaks and a low column backpressure to be obtained, as well as a more sensitive method to be used, compared to other columns.

The mobile phase and gradient were optimized using acetic acid, formic acid trifluoroacetic acid in water (mobile phase A), and methanol or acetonitrile (mobile phase B). Preliminary experiments determined that 0.05% TFA in water and acetonitrile were optimal conditions [30]. The optimized elution, with a total run time of 8 min, consisted of one consecutive acetonitrile gradient step, each with increasing slopes (from 10 to 60% in 5.5 min), and a final 2.4-min re-equilibration time. The column temperature was kept at 25 °C. The detection of catechins was performed at λ = 270 nm.

Figure S1 displays UHPLC–UV chromatogram of catechin standards. The order of the catechins elution is as following, non-epi-forms without gallate GC and C, epi-forms without gallate EGC and EC, epi-forms with gallate EGCG, non-epi-forms with gallate GCG, and finally ECG [31].

### 2.2. SLE Extraction Parameters

#### 2.2.1. Screening of Ionic Liquids, Deep Eutectic Solvents and Conventional Solvents

The ideal solvent type and extraction method for target compounds extracted from the sample was vital for the optimization of the process. High extraction yields can be obtained by decreasing solution viscosity, since at lower viscosity the solvent can easily penetrate the sample matrix. Thus far, for the extraction of catechins from tea samples, several organic solvents such as ethyl acetate, *n*-hexane, and petroleum ether have been used [7].

**Figure 1:**
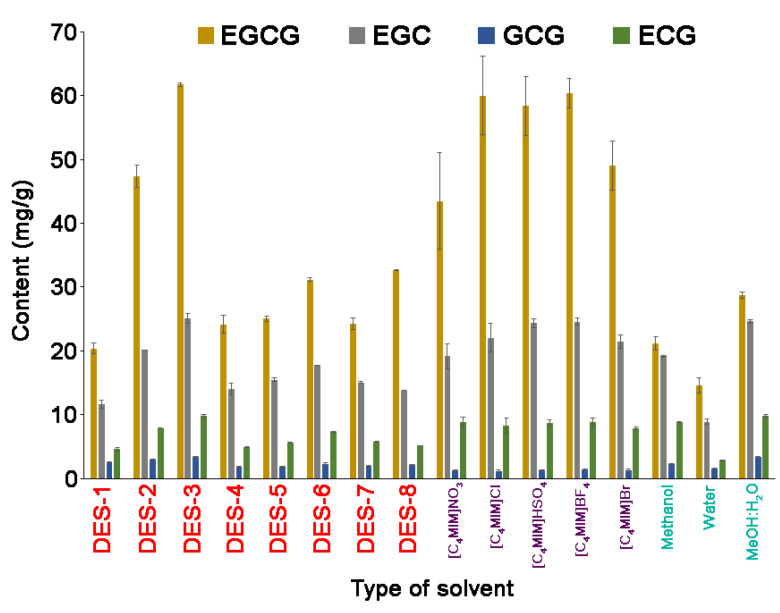
A comparison of the deep eutectic solvents (DESs), ionic liquids (ILs) and conventional solvent extraction efficiency of catechins.

In this study, the extraction efficiency of five ionic liquids ([C_4_MIM]NO_3_, [C_4_MIM]Cl, [C_4_MIM]HSO_4_, [C_4_MIM]BF_4_, [C_4_MIM]Br), eight DESs (containing malic acid, citric acid, and L-lactic acid as hydrogen bond donors) and three conventional solvents (methanol, water, mixture of methanol:water (1:1; *v*/*v*)) were tested for the isolation of selected catechins from tea. The results are shown in Figure 1.

In the extraction of DES-3, comparable yields were observed for the mixture of methanol:water and [C_4_MIM]BF_4_, which were obviously higher than those of methanol, water, [C_4_MIM]NO_3_ and [C_4_MIM]Cl. Solvatochrome test ILs based on tetrafluoroboric acid and DES-3 are characterized as highly polar, which influences the effective extraction of catechins. In addition, [C_4_MIM]BF_4_ and DES-3 have a higher acidity than conventional solvents used for comparison. In the case of malic acid as HBD, in DES, high extraction yields were observed, due to stronger multi-interactions, including *π*–*π*, ionic/charge–charge and hydrogen bonding with targeted compounds. Moreover, the efficiency of the extraction can also be dependent on the role of the HBD:HBA ratio. The evidence emerging from examinations shows that by changing the molar ratio of malic acid:GrT from 1:2 to 2:1 the extraction efficiency of catechins from green tea shows a significant increase. In more acidic solvents (for example DESs based on carboxylic acid), catechins are more stable; therefore, the extraction efficiency is greater compared with water or methanol. Conventional organic solvents are usually volatile and toxic. In consideration of sustainability, biodegradability and pharmaceutically acceptable toxicity, the DES-based extraction method proposed in this study is efficient, non-toxic and eco-friendly, and can be used as a green substitute in the extraction of catechins from tea samples. According to the above results, DES-3 with malic acid:GrT (2:1) was selected as the optimal solvent.

#### 2.2.2. Effect of Water Content in DES

**Figure 2:**
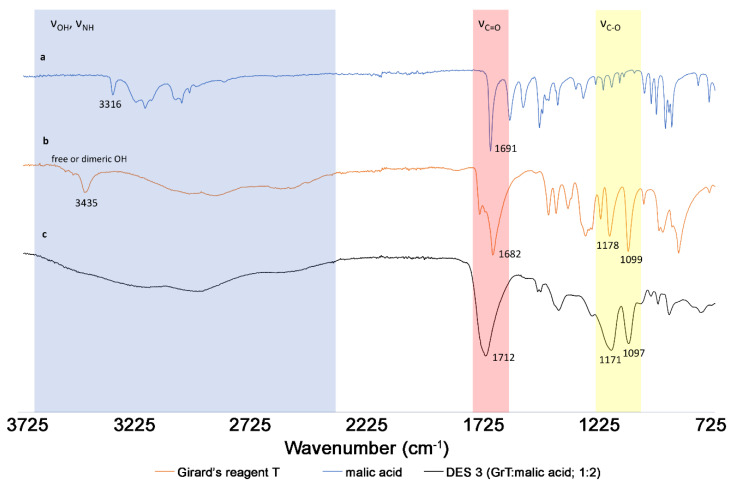
Changes in FT-IR spectra of free components (**a**) Girard’s reagent, (**b**) malic acid and (**c**) the formed DES-3 (GrT:malic acid, 1:2).

After selecting the optimal solvent for catechin extraction, the procedures were performed with DES-3 and different water content (from 10% to 75% (*v*/*v*)). It was found that this factor has a marked impact on extraction yields (Figure S2). A higher water content reduced the viscosity and increased the polarity of the solvent mixture. Moreover, the addition of water affects the structure of eutectic solvents, which can be observed in FT-IR analyses of free-solvent components, DESs, and DESs with different water contents. For example, obtaining DES-3 from free components is related to the formation of a specific supramolecular structure based on hydrogen bonds. This is evidenced by the broadening and shifts of characteristic vibration bands (especially stretching vibrations: ν_OH_, ν_C=O_, ν_C-O_) in the IR spectra (Figure 2).

**Figure 3:**
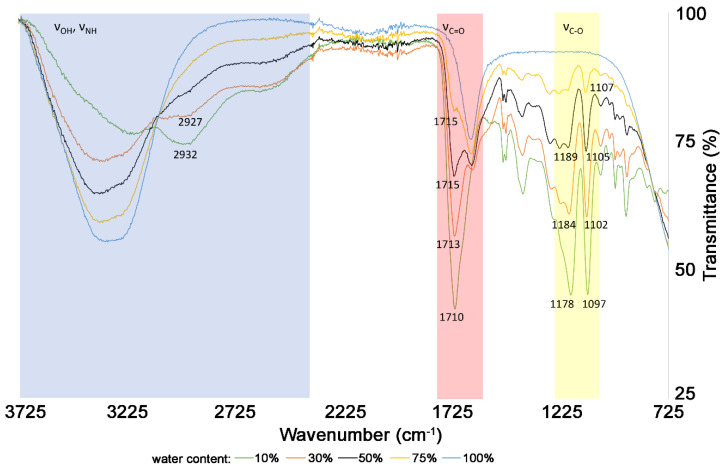
The effect of water content on the supramolecular structure of DES-3 based on FT-IR spectra.

Modifications to the DES-3 structure after water addition are reflected in the FT-IR spectra (Figure 3). According to FT-IR results, upon increasing water content from 30% to 75% (*v*/*v*), significant modifications to the supramolecular structure occur, which decrease the hydrogen bond interconnections between solvent and target bioactive catechins, resulting in the lower extraction yields of analytes.

Therefore, 30% (*v*/*v*) water in DES-3 gave the highest extraction yields and was utilized in further optimization tests.

#### 2.2.3. Effect of Time, Temperature and Solid/Liquid Ratio

In order to optimize certain extraction conditions, response surface methodology (RSM) was adopted using Statistica 12 software package. Central composite design (CCD) was used to conduct the experiments. The effect and interaction of three parameters, specifically extraction time (6.5–73.5 min), temperature (6.5–48.4 °C), and solid/liquid ratio (1:2–1:12), were investigated. Peak area was adopted as the response function.

**Figure 4:**
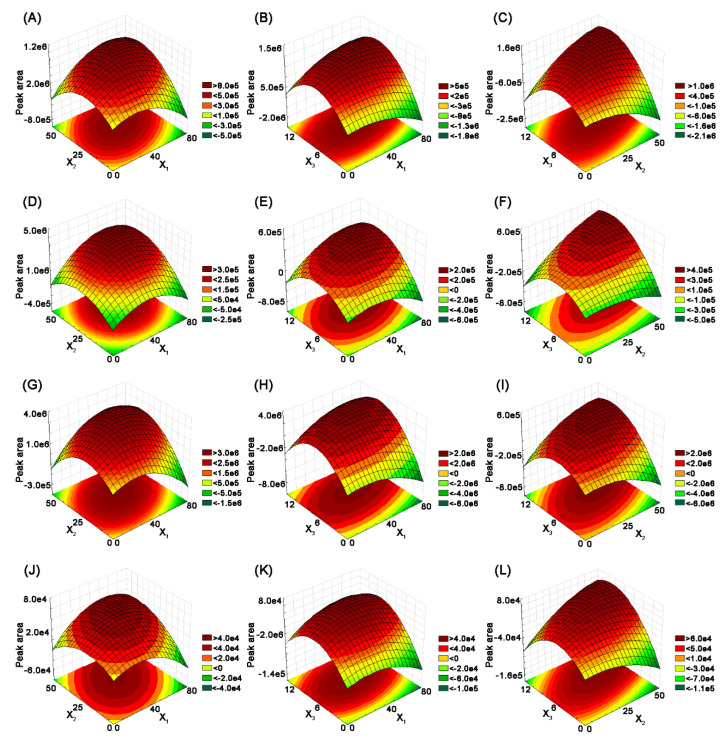
Response surface plots of (**A**, **D**, **G**, **J**) extraction time (X_1_) and temperature (X_2_) at a constant solid/liquid ratio (X_3_) of 1:7, (**B**) extraction time (X_1_) and solid/liquid ratio (X_3_) at a constant extraction temperature (X_2_) of 27.5 °C, (**C**) extraction temperature (X_2_) and solid/liquid ratio (X_3_) at a constant time (X_1_) of 40 min (for (**A**, **B**, **C**)—Epigallocatechin (EGC), (**D**, **E**, **F**)—Epigallocatechin gallate (EGCG), (**G**, **H**, **I**)—Gallocatechin gallate (GCG), (**J**, **K**, **L**)—Epicatechin gallate (ECG)).

For visualization of the obtained results, three-dimensional (3D) RSM was applied. Figure 4 shows 3D plots of the response surface for the extraction efficiency of selected catechins, as related to extraction time (*X*_1_), temperature (*X*_2_), and solid/liquid ratio (X_3_), respectively.

Based on the obtained results, an increase in extraction time from 40 to 50 min and in temperature to 50 °C enhanced the extraction yields of target compounds. When the time was constant at 50 min, and with an increase in temperature and solid/liquid ratio, the extraction yield of catechins increased within a certain range. The mass transfer of analytes from the tea samples to the DES solvent can be easier at higher temperatures, because of the decreased physical adsorption and chemical interactions between analytes and matrices. However, when the solid/liquid ratio and temperature exceed a certain value, the extraction yield declines.

As illustrated in Figure 4, the extraction yields of target compounds significantly increase with an increase in solid/liquid ratio, especially with a short time period. The extraction efficiency of catechins improve the solid/liquid ratio increase to 1:10 and extraction time to 50 min; however, the extraction yields decrease when the liquid/solid ratio exceeds 1:12. The extraction yield of target compounds increases with an increasing extraction temperature over a short extraction time. When the extraction temperature exceeds 50 °C with a longer extraction time, the extraction yield plateaus. Finally, the following DES extraction conditions were selected: extraction time: 50 min; extraction temperature: 50 °C; solid/liquid ratio: 1:10.

ANOVA was performed to evaluate the optimal conditions of DES and the relationship between the response and variables. A second-order polynomial equation for the extraction yield (Y) and variables was obtained by a multiple regression analysis of the experimental data. An ANOVA analysis is shown in Table S5. An F-test was examined in order to investigate the significance of each coefficient, allowing for the determination of the *p*-value. The F-value of lack of fit (*p* = 0.1) was not significant, which supported this model, giving an accurate representation of the experimental data. The coefficient of determination (R^2^) was higher than 0.8721, showing that the experimental data were in accordance with predicted values. Furthermore, Table S5 indicates that linear coefficients (X_1_, X_2_ and X_3_) and cross product coefficients (X_1_X_2_, X_1_X_3_, X_2_X_3_) are considered to be significant (*p* < 0.05), depending on compounds. Based on these results (Table S5), it can be stated that, depending on the compounds, the following effects have a significant impact on the electrochemical conversion efficiency: for EGC X_3_, X_2_, X_3_^2^, X_1_^2^, X_2_X_3_, X_1_X_3_, X_1_, for EGCG: X_3_, X_3_^2^, X_2_X_3_, X_2_^2^, X_1_^2^, X_1_X_3_, for GCG: X_3_, X_3_^2^, X_2_X_3_, X_1_^2^, for ECG: X_3_, X_3_^2^, X_2_X_3_, X_2_^2^, X_1_X_3_, X_2_, X_1_X_2_.

(1) $$Y_{\mathrm{EGC}}=239295+1394X_{1}-90{X_{1}}^{2}+5223X_{2}-280{X_{2}}^{2}+64268X_{3}-7985{X_{3}}^{2}+96.4X_{1}X_{2}+783.7X_{1}X_{3}+1508X_{2}X_{3};R^{2}=0.9383$$

(2) $$Y_{\mathrm{EGCG}}=602911-24536X_{1}-683{X_{1}}^{2}-10429X_{2}-3075{X_{2}}^{2}+807328X_{3}-95278{X_{3}}^{2}+1338X_{1}X_{2}+7245X_{1}X_{3}+18202X_{2}X_{3};R^{2}=0.8940$$

(3) $$Y_{\mathrm{GCG}}=19620+109X_{1}-16{X_{1}}^{2}-212X_{2}-38{X_{2}}^{2}+14103X_{3}-1515{X_{3}}^{2}+21X_{1}X_{2}+94X_{1}X_{3}+271X_{2}X_{3};R^{2}=0.8907$$

(4) $$Y_{\mathrm{ECG}}=88166-5663X_{1}-200{X_{1}}^{2}-6567X_{2}-646{X_{2}}^{2}+216979X_{3}-26344{X_{3}}^{2}+346X_{1}X_{2}+2080X_{1}X_{3}+5259X_{2}X_{3};R^{2}=0.8721$$

As shown in Figure S3, the plot of experimental values for extraction efficiency vs. those calculated from Equations (1)–(4) showed a good fit (i.e., EGC, EGCG, GCG, ECG). As a result of the full factorial design, a Pareto chart was drawn for selected catechins to visualize the estimated effects of the main variables and their interactions. Figure S4 shows the Pareto graphic analysis for selected catechins. The Pareto chart gives a graphical presentation of these effects and it allows for an assessment of both the magnitude and importance of an effect. In the Pareto charts, the bars (variables) that graphically exceed the significance line exert a statistically significant influence on the obtained results.

### 2.3. Validation of Method

The results of the developed UHPLC–UV method were validated in terms of their selectivity, linearity, limits of detection (LOD), limits of quantification (LOQ), precision, accuracy and recovery.

Selectivity was assessed using a matrix comparison method. The results of chromatographic testing showed that the chromatographic peaks of catechins were clearly separated in tea samples. Hence, this method is considered selective and appropriate for the identification and quantitative analysis of catechins in various tested tea samples.

Linear calibration curves were obtained over the range 1–40 µg/g (Table S6) (based on the peak area of analytes, corrected with the IS). All analytes showed good linearity with a coefficient of determination (*R*^2^) ranging from 0.9983 to 0.9990 for the seven catechin standards.

The developed method’s sensitivity was assessed by determining the limits of detection (LOD) and quantification (LOQ). LOD and LOQ were calculated at signal-to-noise ratios (S/N) of 3:1 and 10:1, respectively. LOD values were 0.33 µg/g for each catechin, while LOQ values were 1.0 µg/g for all analytes.

High (HQC = 35 µg/g), medium (MQC = 20 µg/g) and low (LQC = 2.5 µg/g) concentrations of the quality control (QC) samples were analyzed in triplicate within one day (intra-day precision and accuracy) and on three different days (inter-day precision and accuracy). Precision was expressed as the percentage of relative standard deviations (%RSD) and accuracy as the relative error (%RE). As shown in Table S7, RSD and RE obtained by the proposed approach was <11% and between −10–9.8%, respectively.

The recoveries with relative standard deviations (RSDs) for each catechin were measured by spiking blank yellow tea samples in six replicates at three different spiked levels (2.5; 20 and 35 µg/g). The recoveries ranged from 67.6 to 109%, with RSDs of 2.5–8.9% (Table S7).

The validation data showed that the proposed method provides good linearity, sensitivity, selectivity, accuracy, precision and recovery for the simultaneous analysis of seven catechins.

### 2.4. Application of the Developed Method to Real Samples

The new method was applied to analyze nine green, nine black, and two fruit tea samples, which were obtained from Polish markets. In Table 1, we present the obtained results. Due to the high content of catechins in the tested samples, some extracts were diluted (20-, 100- or 200-fold).

**Figure 5:**
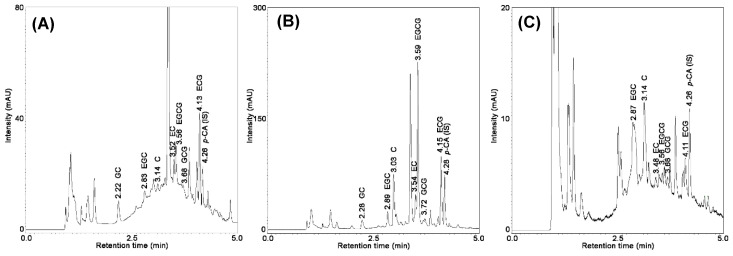
Representative chromatograms obtained for an extract of leaves of (**A**) black tea, (**B**) green tea, and (**C**) fruit tea using the proposed DES–solid–liquid extraction (SLE)–UHPLC–UV method.

The content of catechins in tea varies due to the method of cultivation and treatment of the leaves. The highest level of catechins was determined in green teas, which is consistent with the literature data [8,32]. Their chemical composition is closest to the composition of the fresh tea plant leaves, since in the processing stage, the leaves are not fermented. The lowest concentration of catechins was determined in fruit teas, because they are products generated from dried fruits. Based on the literature data, a high concentration of EGCG, ECG, EGC and EC in tea samples was reported. Interestingly, our research showed that, in each type of tea, a different catechin predominates. In green tea, the highest content of the strongest antioxidant, was EGCG (27.7–63.1 mg/g). In good-quality leaves, this compound makes up 50% of all catechins. In black tea, it was found that the highest content was gallocatechin (6.3–22.8 mg/g), whereas in fruit it was epigallocatechin (8.07–8.4 mg/g). The results indicate that the quantitative differentiation of catechins occurs not only between different species of teas but also for different producers and forms of the same tea. Figure 5 shows UHPLC–UV chromatograms obtained for extracts of leaves of (A) black tea, (B) green tea, and (C) fruit tea. Figure S5 shows the representative multiple reaction monitoring (MRM) chromatograms of the analyzed extracts of green tea.

As part of the study, catechins in tea infusions were also determined. SPE extraction was used to isolate the analytes from the sample, and then analyzed using UHPLC–UV. Catechins were extracted from tea leaves into a water solution to varying degrees. It was estimated that during the traditional method of brewing, approximately 60–70% of compounds found in a dry product passed into the brew. Tested infusions of green, black and fruit tea contained, respectively, 42.5–66.7 mg, 6.9–29.4 mg, 7.5–10.4 mg catechins, in 1 g of product. Based on the results obtained, approximately 50–80% of catechins transfer to the water from the dry product. The content of catechins determined in tea infusions is shown in Table S8.

## 3. Materials and Methods

### 3.1. Chemicals and Reagents

Analytical standards of CA, EC, EGC, ECG, EGCG, GC, GCG, *p*-coumaric acid (*p*-CA, used as internal standard) and Nile red were obtained from Sigma-Aldrich (Steinheim, Germany). Acetonitrile and trifluoroacetic acid (TFA) (HPLC grade) were from Merck (Darmstadt, Germany). Doubly distilled water was prepared using A Milli-Q water purification system (Merck Millipore, Bedford, MA, USA). Choline chloride, L-lactic acid, citric acid and malic acid were bought from Alfa Aesar (Lancashire, UK). Girard’s reagent T (GrT) was obtained from Acros Organics (Geel, Belgium). The ILs, 1-butyl-3-methylimidazolium bromide [C_4_MIM]Br, 1-butyl-3-methylimidazolium chloride [C_4_MIM]Cl, 1-butyl-3-methylimidazolium nitrate [C_4_MIM]NO_3_, 1-butyl-3-methylimidazolium hydrogen sulphate [C_4_MIM]HSO_4_, 1-hexyl-3-methylimidazolium tetrafluoroborate [C_6_MIM]BF_4_, and 1-butyl-3-methylimidazolium hexafluorophosphate [C_4_MIM]PF_6_ were provided by Sigma-Aldrich (St. Louis, MO, USA). Methanol, hydrochloric acid, phosphoric acid and dimethylformamide (DMF) (analytical grade) were from Chempur (Piekary Śląskie, Poland).

Aqueous solutions of ILs (1 M) were prepared by dissolving a precise amount of each IL in deionized water.

A standard solution of each catechin (1 mg/mL) was obtained by dissolving a certain amount of the analytical standard in methanol (10.0 mL). A stock solution of 100 μg/mL, containing all analytes, was prepared by transferring 1.0 mL of each of the seven individual catechin standard solutions into a 10-mL volumetric flask, then adding methanol to the mark. The stock solution was stored in the refrigerator at 5 °C. The working solutions were prepared daily by dilution of the stock solution with the mobile phase (0.05% TFA in water:acetonitrile, 90:10 *v*/*v*).

### 3.2. Synthesis of Deep Eutectic Solvents

DESs based on Girard’s reagent T were prepared according to our previously described ultrasound-assisted method [33].

Two or three components and a calculated amount of deionized water were added to a glass vial, sealed with a screw-cap and exposed to ultrasound (37 kHz, 30 W) at 60 °C until a homogeneous liquid was formed (10–30 min). Eight different DESs, including two or three components, were obtained and examined. The composition, molar ratios, and symbols of DESs used throughout this study are shown in Table S1.

### 3.3. Study of DES Properties

#### 3.3.1. Polarity

The polarity of the obtained DESs was tested in solvatochromatic probe using Nile red (NR). λ_max_ was determined and used in the formula E_NR_ (kcal/mol) = hcλ_maxNA_ = 28591/λ_max_ to obtain ENR [22].

The obtained results of DES polarity with 30% of water are summarized in Table S2. DES 8, DES 3 and DES 4 are the most polarized (ca. 47 kcal/mol), with polarities similar to water (46.9 kcal/mol). DES 5, DES 7 and DES 1 are the least polarized, with polarities similar to MeOH (51.8 kcal/mol).

#### 3.3.2. Density and Viscosity

Viscosity tests of eight prepared DESs were carried out using a DV1 Viscometer (Brookfield, Middleboro, MA, USA) at 27 °C. A portable density meter, Densito 30PX (Mettler Toledo, Schwerzenbach, Switzerland), was used for density analysis at 27 °C. The obtained results are summarized in Table S2. No specific correlations between the efficiency of the extraction and the density or viscosity of the tested DESs are observed.

#### 3.3.3. IR Spectroscopy

IR spectra were measured on the FT-IR spectrometer Nicolet 6700 at room temperature using the ATR method (Thermo Fisher Scientific, Waltham, MA, USA). The spectra of Girard’s reagent, malic acid, their eutectic mixture and eutectic mixture with different water contents (10, 30, 50 and 75%) were recorded and compared.

### 3.4. Extraction of Catechins from Tea Samples

#### 3.4.1. Comparison of Extraction Procedures

For the purpose of comparison, extractions employ: (1) conventional solvents (water, methanol and mixture water:methanol (1:1; *v*/*v*)); (2) ionic liquids ([C_4_MIM]Br, [C_4_MIM]Cl, [C_4_MIM]NO_3_, [C_4_MIM]HSO_4_, [C_6_MIM]BF_4_, [C_4_MIM]PF_6_); (3) DESs (Table S1) were performed. Briefly, the ground tea leaves (150 mg) were weighed, then the extraction solvent (1.5 mL) was added and the sample was stirred at 1100 rpm for 40 min at 40 °C in an Eppendorf tube using thermomixer comfort (Eppendorf AG, Hamburg, Germany). Then, the sample was centrifuged for 5 min at 2000× *g* (IKA mini G centrifuge, Staufen, Germany) and the liquid supernatant (600 µL) was transferred to another tube. The extract was diluted 1:1 with methanol. The final extract was filtered through a 0.2-µm nylon membrane syringe filter and transferred to a vial for UHPLC–UV analysis.

#### 3.4.2. Optimization of DES–SLE Extraction Procedure

Firstly, during the optimization of the DES–SLE extraction procedure, the influence of water the content in DESs was investigated by considering four values: 10%, 30%, 50% and 75% of water in DES.

In the presented study, to optimize the extraction conditions (temperature, time of extraction and solid/liquid ratio), the response surface methodology (RSM) with three-factor and rotatable central composite design (CCD) was applied [34]. The design variables were extraction time (6.5–73.5 min; *X*_1_), temperature (6.5–48.4 °C; *X*_2_) and liquid-to-solid ratio: 1:2–1:12; mg/mL; *X*_3_).

The generated runs are shown in Table S3. Randomizing the order of the experiments was applied to minimize the influences of unexplained variability in the observed response, caused extraneous factors. Multiple linear regression analysis was performed using Statistica 12 software (StatSoft, Krakow, Poland). The experimental data were fitted to the second-order polynomial model (Equation (5)) and regression coefficients (*β*s) were obtained.

(5) $$Y=\beta_{0}+\sum_{i=1}^{k}\beta_{i}X_{i}+\sum_{i=1}^{k}\beta_{ii}X_{i}^{2}+\sum_{\begin{array}{l} i=1\\ i<j \end{array}}^{k-1}\sum_{j=2}^{k}\beta_{ij}X_{i}X_{j}$$

where *X*_1_, *X*_2_, …, *X_k_* are the independent variables affecting the responses *Y*′s; *β_0_*, *β_i_* (*i* = 1, 2, …, *k*), *β_ii_* (*i* = 1, 2, …, *k*), and *β_ij_* (*i* = 1, 2, …, *k*; *j* = 1, 2, …, *k*) are the regression coefficients for intercept, linear, quadratic, and interaction terms, respectively; *k* is the number of variables.

All optimization procedures were carried out in triplicate. The selection of optimal conditions was based on the peak area obtained for selected catechins (EGC, EGCG, ECG, GCG).

### 3.5. Instrumentation and Chromatographic Conditions

A Hitachi Elite LaChrom UHPLC system coupled with a UV detector was used for chromatographic analysis (Merck Hitachi, Darmstadt, Germany). Chromatographic separation was achieved using Poroshell 120 SB-C18 (100 × 4.6 mm, 2.7 μm) from Agilent. A binary mobile phase was used for the chromatographic separation, comprised of 0.05% trifluoroacetic in water (solvent A) and acetonitrile (solvent B). The gradient elution started at 10% B and increased to 60% over 5.5 min, after which it decreased to 10% in 0.1 min, and was finally allowed to stabilize for 2.4 min; thus, the overall runtime was 8 min. The injection volume was 2 μL, the flow rate was fixed at 1.0 mL/min, and the separation was performed at 25 °C. The absorbance of all analytes was measured at λ = 270 nm.

Individual compounds were identified by comparing their retention times using the standard addition method.

A Dionex UHPLC system (Dionex Corporation, Sunnyvale, CA, USA) with an AB Sciex Q-Trap^®^ 4000 mass spectrometer (Foster City, CA, USA) was used to confirm the presence of the determined catechins. The chromatographic conditions of UHPLC–MS/MS were the same as in the UHPLC–UV method, aside from the application of TFA in water as a component of the mobile phase. TFA was replaced by the 0.1% formic acid.

Electrospray ionization (ESI) conditions in the positive mode were first optimized with direct infusion into the mass spectrometer to select the precursor and product ions resulting from fragmentation, declustering potential (DP) and collision energy (CE) for each catechin (Table S4). The catechins were evaluated by employing multiple reaction monitoring (MRM) mode (Table S4). The other working parameters of the mass spectrometer were as follows: curtain gas 20 psi (nitrogen), collision gas-medium, ion spray voltage −4500 V, temperature 500 °C, ion source gases nebulizer gas 60 psi/auxiliary gas 50 psi (both nitrogen), entrance potential −5 V. Additionally, the dwell times of the analytes were set to 50 ms. Equipment control and data acquisition were performed with Analyst 1.5.1 software (Applied Biosystems, Foster City, CA, USA).

### 3.6. Analysis of Catechins in Tea Samples

In the quantitative analysis of tea, 9 samples of black tea (Assam), 9 samples of green tea (Long Jing, Bi Luo Chun, Yu Hua Cha, Jasmine, Sencha) and 2 samples of fruit tea were used in this study. All tea products were purchased from local markets in Poland. In total, 20 teas, cultivated in four countries (China, Japan, India, Vietnam), were used in this study. The origin of the teas was guaranteed by the seller. Blended products were used in this study (fruit tea).

Leaves of the teas were ground with a mill, 30 μL of the *p*-CA (internal standard; 50 µg/mL) was added and then samples were extracted using the following DES–SLE procedure: a 150-mg tea sample was added to a 2 mL Eppendorf tube and 1.5 mL of 30% DES-3 (malic acid:GrT; 2:1; *v*/*v*) was added. The sample was stirred at 50 °C for 50 min at 1100 rpm using thermomixer. Then, the sample was centrifuged at 1725 rpm for 5 min, the supernatant decanted, which was transferred to another tube, and 600 µL of the extract was diluted with 600 µL methanol. Thus, the mixture solvent was ready for UHPLC–UV analysis after being passed through a 0.22 μm nylon filter.

Additionally, catechins were extracted from tea infusions. A moderate to strong brew of black tea was prepared following the instructions provided with the 0.15 g tea by pouring 15 mL of boiling water into a beaker and dipping a teabag for 2–3 min. Green tea was prepared in water at a temperature of 80–90 °C. Next, all infusion tea samples were extracted based on the SPE procedure described in [35]. Samples were extracted using a solid-phase extraction (SPE) system (BAKERBOND spe-12G system, J.T. Baker Inc., Deventer, Netherlands). The cartridges (Oasis HLB, Waters) were conditioned with 3 mL of water (adjusted to pH = 3.5 with hydrochloric acid), then 3 mL 70% DMF with 0.1% phosphoric acid and 3 mL of water (adjusted to pH = 3.5 with hydrochloric acid). In the proposed method, 15 mL of a tea sample was adjusted to pH = 3.5 with hydrochloric acid and then loaded onto the cartridges. The catechins were eluted with 5 mL 70% DMF with 0.1% phosphoric acid. Finally, the eluate was injected into the UHPLC–UV system. The identity of the catechins in the leaves and infusion samples was confirmed by the UHPLC–MS/MS method in MRM mode.

## 4. Conclusions

The extraction of catechins from tea samples using a novel malic acid-based DES method demonstrated, for the first time, that DES-type solvents have promising prospects in the recovery of bioactive substances.

The extraction method using DES-3 (malic acid:GrT, 2:1) was significantly more efficient for extraction of seven main catechins than the previous time-consuming methods that employed organic solvents. The optimal performance was obtained at a temperature of 50 °C, a time of 50 min, the extraction solvent malic acid:GrT with a 2:1 M ratio and 30% water content, and the solid/liquid ratio 1:10 mg/mL. The effective UHPLC–UV method revealed excellent precision, accuracy, and recovery, and was applied to the determination of seven catechins in tea samples. We have shown the applicability of DES as an extraction solvent; however, due to its unique advantages, such as its environmentally benign behavior, low cost and non-toxicity, it holds great promise in other areas. In conclusion, DES–SLE provided a promising strategy to extract active compounds from tea samples for potential applications.

## Figures and Tables

**Table 1:** Content of catechins determined in leaves of tea samples.

Sample		Concentration (mg/g of Dry Weight ^a^)						
		GC	EGC	C	EC	EGCG	GCG	ECG
**Black Tea**	**1**	10.2 ± 0.77 ^b^	4.18 ± 0.56	1.68 ± 0.13	1.70 ± 0.20	4.51 ± 0.12	1.06 ± 0.83	6.37 ± 0.55
	**2**	17.1 ± 1.50	3.39 ± 0.33	0.93 ± 0.01	2.15 ± 0.20	3.68 ± 0.19	0.22 ± 0.02	6.10 ± 0.11
	**3**	13.4 ± 0.03	3.47 ± 0.27	1.15 ± 0.06	1.25 ± 0.11	4.57 ± 0.34	0.53 ± 0.07	4.56 ± 0.48
	**4**	17.5 ± 0.15	7.35 ± 0.97	0.77 ± 0.04	1.90 ± 0.18	5.40 ± 0.24	0.52 ± 0.02	7.03 ± 0.50
	**5**	22.8 ± 1.08	5.41 ± 0.08	0.98 ± 0.01	1.50 ± 0.15	5.18 ± 0.23	0.37 ± 0.01	6.74 ± 0.17
	**6**	6.34 ± 0.13	3.76 ± 0.07	1.35 ± 0.02	2.05 ± 0.05	8.91 ± 0.23	0.24 ± 0.03	4.68 ± 0.10
	**7**	5.80 ± 0.08	2.25 ± 0.19	1.41 ± 0.01	4.29 ± 0.19	1.94 ± 0.04	0.89 ± 0.02	4.03 ± 0.02
	**8**	9.67 ± 0.05	0.65 ± 0.05	0.70 ± 0.03	0.34 ± 0.03	0.37 ± 0.06	0.40 ± 0.02	0.96 ± 0.03
	**9**	6.64 ± 0.08	1.17 ± 0.02	0.74 ± 0.06	0.44 ± 0.04	0.34 ± 0.04	0.39 ± 0.02	0.87 ± 0.01
**Green Tea**	**10**	5.99 ± 0.46	18.7 ± 0.39	0.46 ± 0.07	3.87 ± 0.31	41.0 ± 0.06	5.86 ± 0.25	9.35 ± 0.42
	**11**	9.58 ± 0.29	15.9 ± 0.43	1.07 ± 0.03	3.17 ± 0.23	41.2 ± 1.64	5.68 ± 0.09	11.0 ± 0.45
	**12**	17.6 ± 0.45	21.5 ± 0.73	3.43 ± 0.13	2.84 ± 0.12	42.1 ± 2.80	3.72 ± 0.10	14.4 ± 0.73
	**13**	9.90 ± 0.65	17.1 ± 0.05	2.37 ± 0.10	3.60 ± 0.08	27.2 ± 0.45	1.86 ± 0.02	17.0 ± 0.48
	**14**	7.09 ± 0.11	12.4 ± 0.34	1.75 ± 0.02	8.21 ± 0.45	46.8 ± 2.29	8.13 ± 0.07	9.91 ± 0.20
	**15**	7.30 ± 0.32	17.1 ± 0.18	2.59 ± 0.05	1.85 ± 0.16	53.8 ± 0.95	4.42 ± 0.08	15.5 ± 1.03
	**16**	11.5 ± 0.93	10.9 ± 0.48	3.64 ± 0.11	1.39 ± 0.17	53.5 ± 2.25	3.75 ± 0.01	11.2 ± 0.15
	**17**	5.80 ± 0.11	18.5 ± 0.31	1.10 ± 0.03	0.84 ± 0.06	63.1 ± 1.04	5.36 ± 0.02	11.1 ± 0.34
	**18**	19.8 ± 0.06	23.4 ± 1.07	0.86 ± 0.01	1.28 ± 0.05	53.2 ± 0.68	6.44 ± 0.13	8.01 ± 0.21
**Fruit Tea**	**19**	ND ^c^	8.40 ± 0.01	3.55 ± 0.04	0.26 ± 0.02	0.16 ± 0.04	0.16 ± 0.04	0.23 ± 0.01
	**20**	ND	8.07 ± 0.08	2.67 ± 0.07	0.25 ± 0.02	0.21 ± 0.06	ND	0.20 ± 0.01

^a^ Each value is the mean (µg/g of dry weight) of three replications; ^b^ SD relative standard deviation; ^c^ not detectable (ND).

## Supplementary Materials

The following are available online. Figure S1. UHPLC–UV chromatogram of a standard solution containing the analyzed catechins and IS, Figure S2. Effect of water content in DESs on extraction efficiency of catechins, Figure S3. The experimental data vs. predicted data for extraction efficiency of the conversion of (A) EGC, (B) EGCG, (C) GCG, (D) ECG, Figure S4. Pareto chart showing the values of effects from variables using the extraction efficiency of (A) EGC, (B) EGCG, (C) GCG, (D) ECG, Figure S5. Representative TIC-MRM chromatogram obtained for an extract of leaves of green tea after DES–SLE procedure using UHPLC–MS/MS method, Table S1. List of tested deep eutectic solvents (DES), Table S2. Polarity, viscosity and density of tested deep eutectic solvents (DESs), Table S3. Rotatable central composite design setting in the original and coded form of the independent variables (*X*_1_, *X*_2_ and *X*_3_), Table S4. MRM transitions and mass spectrometer parameters, Table S5. Analysis of variance (ANOVA) for fit of extraction efficiency from central composite design, Table S6. Summary of calibration curve and linearity range of catechins (*n* = 6). Table S7. Summary of accuracy, precision and recovery of catechins (*n* = 6). Table S8. Content of catechins determined in infusion of tea.
