# Supplementary material for: Application of Deep Eutectic Solvents and Ionic Liquids in the Extraction of Catechins from Tea
Source: Molecules. 2020 Jul 14;25(14):3216. doi: 10.3390/molecules25143216 (PMC7397009; doi:10.3390/molecules25143216)
Supplement: Supplementary file 1 [file molecules-25-03216-s001.pdf]

Application of deep eutectic solvents and ionic liquids in the extraction of catechins from tea

Sylwia Bajkacz[1,2,], Jakub Adamek[2,3], Anna Sobska[1]*

[1] Department of Inorganic, Analytical Chemistry and Electrochemistry, Faculty of Chemistry, Silesian University of Technology, Krzywoustego 6, 44-100 Gliwice, Poland

[2] Biotechnology Center of Silesian University of Technology, Krzywoustego 8, 44-100 Gliwice, Poland

[3] Department of Organic and Bioorganic Chemistry and Biotechnology, Faculty of Chemistry, Silesian University of Technology, Krzywoustego 4, 44-100 Gliwice, Poland

SUPPLEMENTARY MATERIALS

Summary

This supporting information file includes additional results and information as described in the text of the main article including:

Figure S1. UHPLC-UV chromatogram of a standard solution containing the analyzed catechins and IS.

Figure S2. Effect of water content in DESs on extraction efficiency of catechins.

Figure S3. The experimental data vs predicted data for extraction efficiency of the conversion of (A) EGC, (B) EGCG, (C) GCG, (D) ECG.

Figure S4. Pareto chart showing the values of effects from variables using the extraction efficiency of (A) EGC, (B) EGCG, (C) GCG, (D) ECG.

Figure S5. Representative TIC-MRM chromatogram obtained for an extract of leaves of green tea after DES-SLE procedure using UHPLC-MS/MS method.

Table S1. List of tested deep eutectic solvents (DES).

Table S2. Polarity, viscosity and density of tested deep eutectic solvents (DESs).

Table S3. Rotatable central composite design setting in the original and coded form of the independent variables (X_1, X_2 and X_3).

Table S4. MRM transitions and mass spectrometer parameters.

Table S5. Analysis of variance (ANOVA) for fit of extraction efficiency from central composite design.

Table S6. Summary of calibration curve and linearity range of catechins (n=6).

Table S7. Summary of accuracy, precision and recovery of catechins (n=6).

Table S8. Content of catechins determined in infusion of tea.

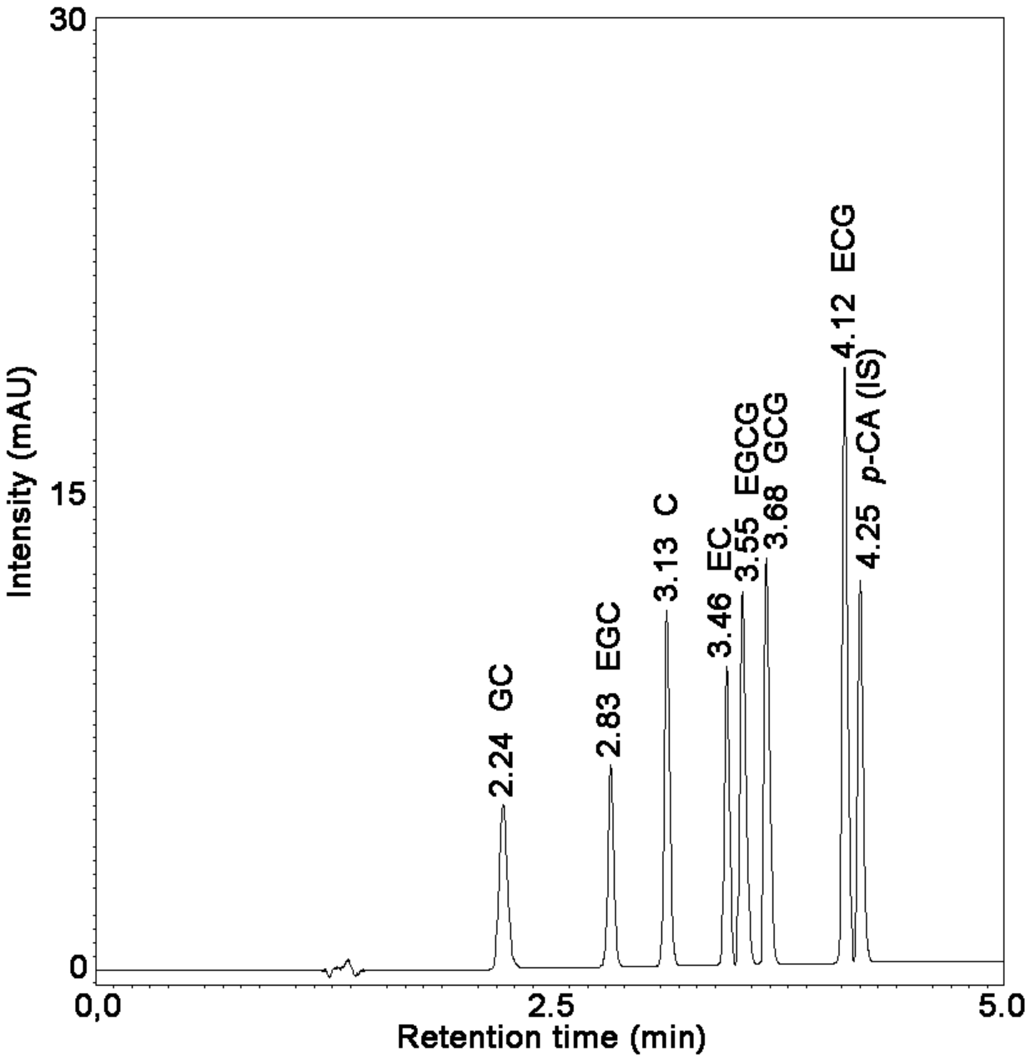

Figure S1. UHPLC-UV chromatogram of a standard solution containing the analyzed catechins (concentration corresponding GC, EGC, C, EC 5 µg/g and EGCG, GCG and ECG 10 µg/g) and IS (concentration corresponding 10 µg/g).

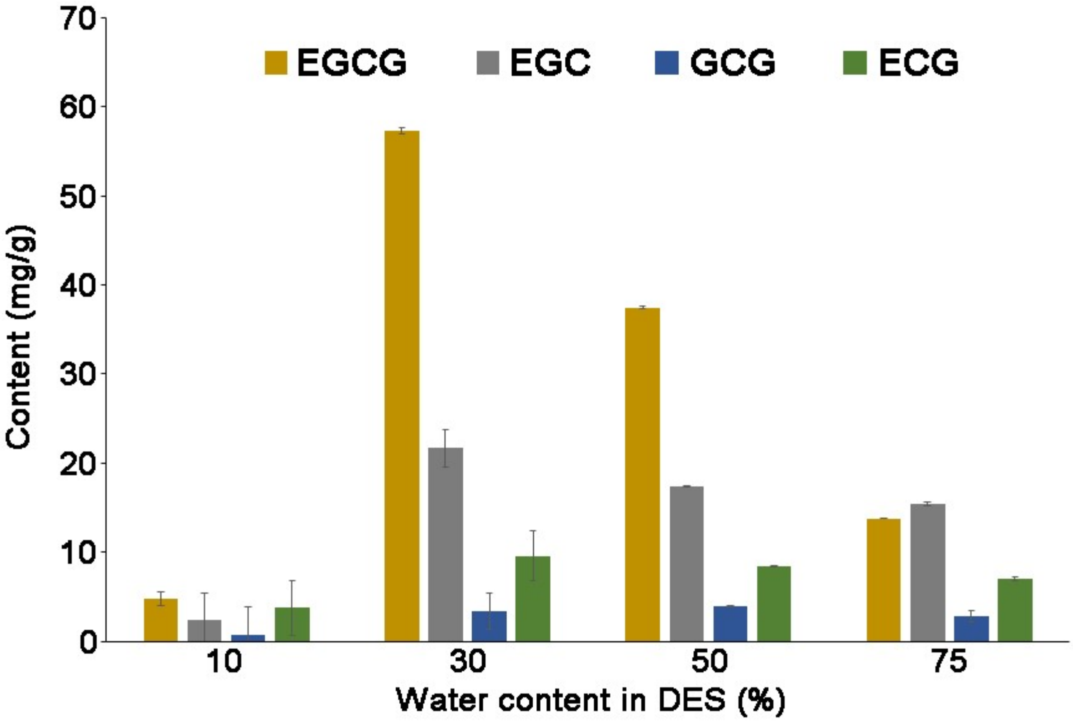

Figure S2. Effect of water content in DESs on extraction efficiency of catechins.

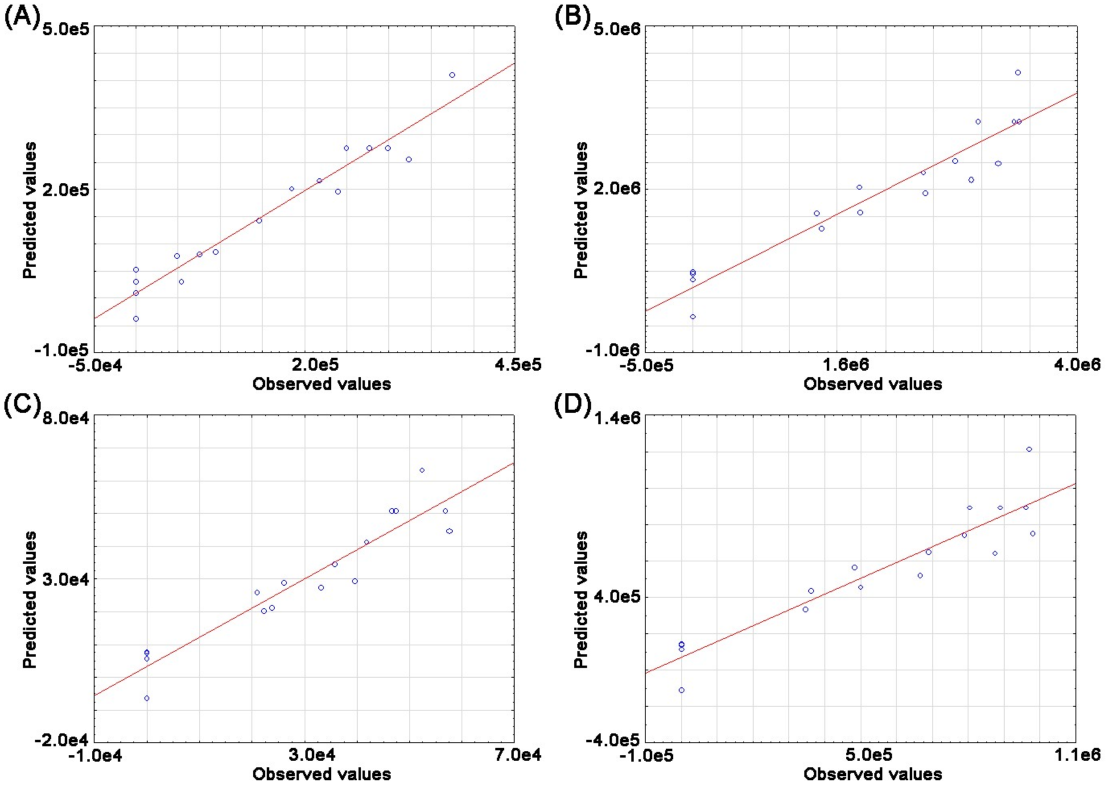

Figure S3. The experimental data vs predicted data for extraction efficiency of the conversion of (A) EGC, (B) EGCG, (C) GCG, (D) ECG.

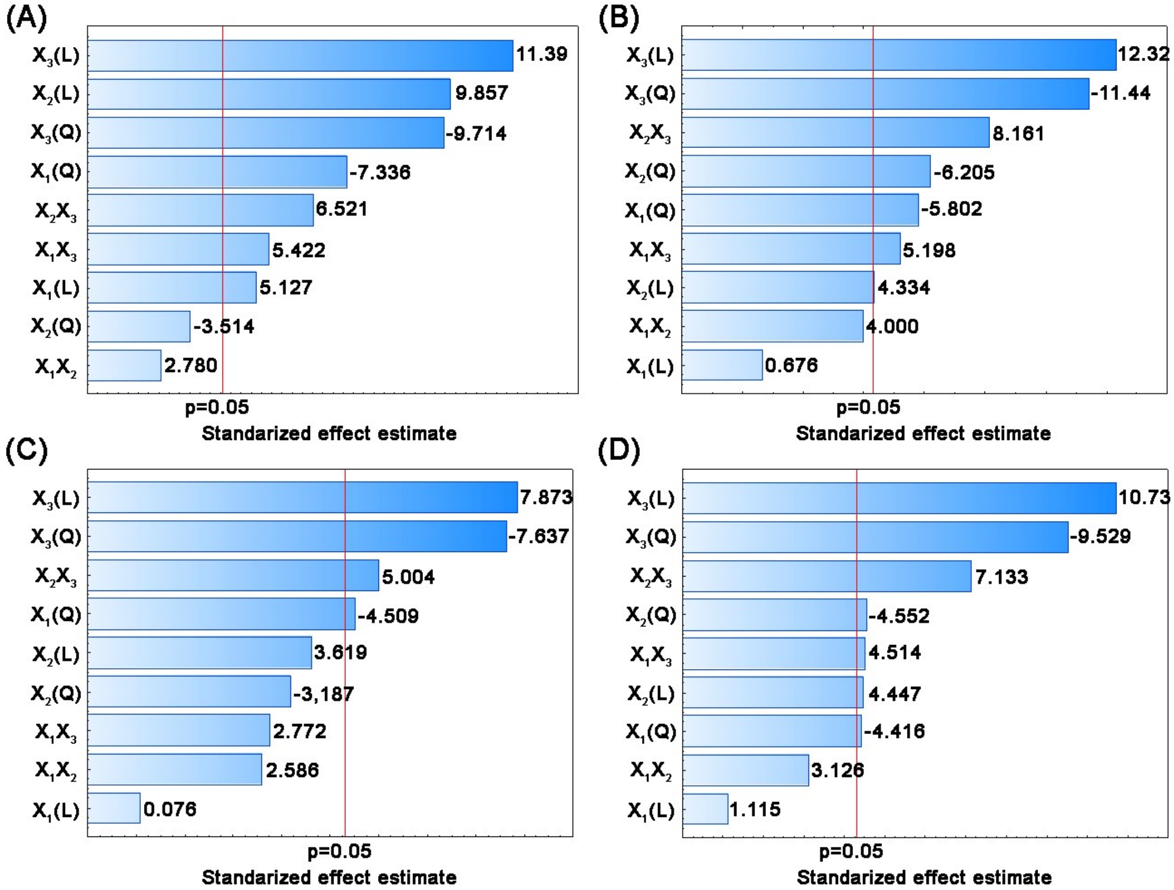

Figure S4. Pareto chart showing the values of effects from variables using the extraction efficiency of (A) EGC, (B) EGCG, (C) GCG, (D) ECG.

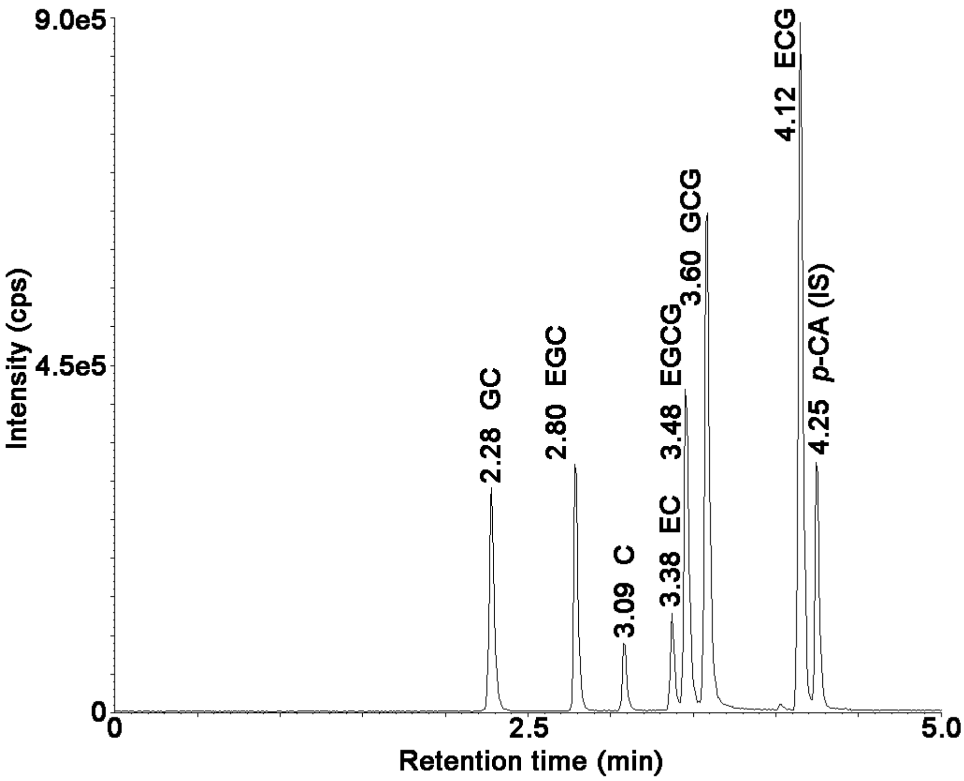

Figure S5. Representative TIC-MRM chromatogram obtained for an extract of leaves of green tea after DES-SLE procedure using UHPLC-MS/MS method.

Table S1. List of tested deep eutectic solvents (DES).

Number	DES			Mole ratio
	**Component 1**	**Component 2**	**Component 3**	
DES-1	L-lactic acid	Girard's reagent	–	2:1
DES-2	citric acid	Girard's reagent	–	2:1
DES-3	malic acid	Girard's reagent	–	2:1
DES-4	malic acid	Girard's reagent	–	1:1
DES-5	malic acid	Girard's reagent	–	1:2
DES-6	malic acid	Girard's reagent	L-lactic acid	1:1:1
DES-7	malic acid	Girard's reagent	choline chloride	1:1:1
DES-8	malic acid	Girard's reagent	citric acid	1:1:1

Table S2. Polarity, viscosity and density of tested deep eutectic solvents (DESs).

**Solvent**	**Polarity (kcal/mol)**	**Viscosity (27°C) mPa·s**	**Density (27°C) g/cm^3**
DES-1	49.15	13.63	1.169
DES-2	48.08	70.64	1.306
DES-3	47.35	29.10	1.267
DES-4	47.46	30.90	1.241
DES-5	49.42	33.71	1.220
DES-6	48.02	20.11	1.221
DES-7	49.13	17.61	1.182
DES-8	47.21	40.63	1.288

Table S3. Rotatable central composite design setting in the original and coded form of the independent variables (X_1, X_2 and X_3).

Experiment	(X_1) Time (min)	(X_2) Temperature (°C)	(X_3) Solid/liquid ratio (mg/mL)
1	6.5	27.5	1:7
2	20	15	1:4
3	20	15	1:10
4	20	40	1:4
5	20	40	1:10
6	40	6.5	1:7
7	40	27.5	1:2
8	40 (C)	27.5	1:7
9	40 (C)	27.5	1:7
10	40 (C)	27.5	1:7
11	40	27.5	1:12
12	40	48.4	1:7
13	60	15	1:4
14	60	15	1:10
15	60	40	1:4
16	60	40	1:10
17	73.5	27.5	1:7

Table S4. MRM transitions and mass spectrometer parameters.

Compound	*Q*1[a] (*m*/*z*)	*Q*2[b] (*m*/*z*)	DP[c] (V)	CE[e] (V)	CXP[f] (V)
**GC**	304.8	124.8	-90	-30	-7
**EGC**	304.8	124.8	-95	-30	-7
**C**	288.9	108.6	-70	-36	-3
**EC**	288.9	108.6	-70	-34	-3
**EGCG**	456.9	168.9	-75	-26	-3
**GCG**	456.9	168.8	-95	-27	-7
**ECG**	441.0	168.9	-75	-26	-3
***p*-CA (IS)**	162.9	118.6	-50	-18	-5

[a] Q1, precursor ion
[b] Q2, fragment ion
[c] DP, declustering potential
[d] EP, entrance potential
[e] CE, collision energy
[f] CXP, cell exit potential

Table S5. Analysis of variance (ANOVA) for fit of extraction efficiency from central composite design.

Source of variation	Sum of square	Df[a]	Mean square	*F*-value[b]	*P*-value
**EGC**					
X_1	$2.93 \cdot 10^{10}$	1	$2.93 \cdot 10^{10}$	$4.86 \cdot 10^{1}$	$1.99 \cdot 10^{-2}$
X_1^2	$2.59 \cdot 10^{10}$	1	$2.59 \cdot 10^{10}$	$4.30 \cdot 10^{1}$	$2.24 \cdot 10^{-2}$
X_2	$1.10 \cdot 10^{11}$	1	$1.10 \cdot 10^{11}$	$1.83 \cdot 10^{2}$	$5.39 \cdot 10^{-3}$
X_2^2	$5.90 \cdot 10^{7}$	1	$5.90 \cdot 10^{7}$	$9.81 \cdot 10^{-2}$	$7.84 \cdot 10^{-1}$
X_3	$7.81 \cdot 10^{10}$	1	$7.80 \cdot 10^{10}$	$1.29 \cdot 10^{2}$	$7.62 \cdot 10^{-3}$
X_3^2	$7.80 \cdot 10^{10}$	1	$7.80 \cdot 10^{10}$	$1.29 \cdot 10^{2}$	$7.62 \cdot 10^{-3}$
X_1X_2	$4.65 \cdot 10^{9}$	1	$4.65 \cdot 10^{9}$	7.73	$1.09 \cdot 10^{-1}$
X_1X_3	$1.77 \cdot 10^{10}$	1	$1.77 \cdot 10^{10}$	$2.94 \cdot 10^{1}$	$3.24 \cdot 10^{-2}$
X_2X_3	$2.56 \cdot 10^{10}$	1	$2.56 \cdot 10^{10}$	$4.25 \cdot 10^{1}$	$2.27 \cdot 10^{-2}$
**Lack of fit**	$1.09 \cdot 10^{11}$	5	$2.19 \cdot 10^{10}$	$3.64 \cdot 10^{1}$	$2.69 \cdot 10^{-2}$
**Pure error**	$1.20 \cdot 10^{9}$	2	$6.01 \cdot 10^{8}$		
**Total SS**	$4.71 \cdot 10^{11}$	16			
**EGCG**					
X_1	$2.56 \cdot 10^{10}$	1	$2.56 \cdot 10^{10}$	$4.57 \cdot 10^{-1}$	$5.69 \cdot 10^{-1}$
X_1^2	$1.88 \cdot 10^{12}$	1	$1.88 \cdot 10^{12}$	$3.37 \cdot 10^{1}$	$2.84 \cdot 10^{-2}$
X_2	$1.05 \cdot 10^{12}$	1	$1.05 \cdot 10^{12}$	$1.88 \cdot 10^{1}$	$4.93 \cdot 10^{-2}$
X_2^2	$2.16 \cdot 10^{12}$	1	$2.16 \cdot 10^{12}$	$3.85 \cdot 10^{1}$	$2.50 \cdot 10^{-2}$
X_3	$8.49 \cdot 10^{12}$	1	$8.49 \cdot 10^{12}$	$1.52 \cdot 10^{2}$	$6.53 \cdot 10^{-3}$
X_3^2	$7.32 \cdot 10^{12}$	1	$7.32 \cdot 10^{12}$	$1.31 \cdot 10^{2}$	$7.56 \cdot 10^{-3}$
X_1X_2	$8.95 \cdot 10^{11}$	1	$8.95 \cdot 10^{11}$	$1.60 \cdot 10^{1}$	$5.72 \cdot 10^{-2}$
X_1X_3	$1.51 \cdot 10^{12}$	1	$1.51 \cdot 10^{12}$	$2.70 \cdot 10^{1}$	$3.51 \cdot 10^{-2}$
X_2X_3	$3.73 \cdot 10^{12}$	1	$3.73 \cdot 10^{12}$	$6.66 \cdot 10^{1}$	$1.47 \cdot 10^{-2}$
**Lack of fit**	$2.70 \cdot 10^{12}$	5	$5.41 \cdot 10^{11}$	9.67	$9.64 \cdot 10^{-2}$
**Pure error**	$1.12 \cdot 10^{11}$	2	$5.60 \cdot 10^{10}$		
**Total SS**	$2.66 \cdot 10^{13}$	16			
**GCG**					
X_1	$1.92 \cdot 10^{5}$	1	$1.92 \cdot 10^{5}$	$5.84 \cdot 10^{-3}$	$9.46 \cdot 10^{-1}$
X_1^2	$6.70 \cdot 10^{8}$	1	$6.70 \cdot 10^{8}$	$2.03 \cdot 10^{1}$	$4.58 \cdot 10^{-2}$
X_2	$4.32 \cdot 10^{8}$	1	$4.32 \cdot 10^{8}$	$1.31 \cdot 10^{1}$	$6.86 \cdot 10^{-2}$
X_2^2	$3.35 \cdot 10^{8}$	1	$3.35 \cdot 10^{8}$	$1.02 \cdot 10^{1}$	$8.59 \cdot 10^{-2}$
X_3	$2.04 \cdot 10^{9}$	1	$2.04 \cdot 10^{9}$	$6.20 \cdot 10^{1}$	$1.58 \cdot 10^{-2}$
X_3^2	$1.92 \cdot 10^{9}$	1	$1.92 \cdot 10^{9}$	$5.83 \cdot 10^{1}$	$1.67 \cdot 10^{-2}$
X_1X_2	$2.20 \cdot 10^{8}$	1	$2.20 \cdot 10^{8}$	6.69	$1.23 \cdot 10^{-1}$
X_1X_3	$2.53 \cdot 10^{8}$	1	$2.53 \cdot 10^{8}$	7.69	$1.09 \cdot 10^{-1}$
X_2X_3	$8.25 \cdot 10^{8}$	1	$8.25 \cdot 10^{8}$	$2.50 \cdot 10^{1}$	$3.77 \cdot 10^{-2}$
**Lack of fit**	$6.57 \cdot 10^{8}$	5	$1.31 \cdot 10^{8}$	3.99	$2.12 \cdot 10^{-1}$
**Pure error**	$6.59 \cdot 10^{7}$	2	$3.29 \cdot 10^{7}$		
**Total SS**	$6.62 \cdot 10^{9}$	16			
**ECG**					
X_1	$7.61 \cdot 10^{9}$	1	$7.61 \cdot 10^{9}$	1.24	$3.81 \cdot 10^{-1}$
X_1^2	$1.19 \cdot 10^{11}$	1	$1.19 \cdot 10^{11}$	$1.95 \cdot 10^{1}$	$4.76 \cdot 10^{-2}$
X_2	$1.21 \cdot 10^{11}$	1	$1.21 \cdot 10^{11}$	$1.98 \cdot 10^{1}$	$4.70 \cdot 10^{-2}$

X_2^2	$1.27 \cdot 10^{11}$	1	$1.27 \cdot 10^{11}$	$2.07 \cdot 10^{1}$	$4.50 \cdot 10^{-2}$
X_3	$7.04 \cdot 10^{11}$	1	$7.04 \cdot 10^{11}$	$1.15 \cdot 10^{2}$	$8.57 \cdot 10^{-3}$
X_3^2	$5.55 \cdot 10^{11}$	1	$5.55 \cdot 10^{11}$	$9.08 \cdot 10^{1}$	$1.08 \cdot 10^{-2}$
X_1X_2	$5.97 \cdot 10^{11}$	1	$5.97 \cdot 10^{10}$	9.77	$8.89 \cdot 10^{-2}$
X_1X_3	$1.25 \cdot 10^{11}$	1	$1.25 \cdot 10^{11}$	$2.04 \cdot 10^{1}$	$4.57 \cdot 10^{-2}$
X_2X_3	$3.11 \cdot 10^{11}$	1	$3.11 \cdot 10^{11}$	$5.09 \cdot 10^{1}$	$1.91 \cdot 10^{-2}$
**Lack of fit**	$2.69 \cdot 10^{11}$	5	$5.38 \cdot 10^{10}$	8.79	$1.05 \cdot 10^{-1}$
**Pure error**	$1.22 \cdot 10^{10}$	2	$6.11 \cdot 10^{9}$		
**Total SS**	$2.20 \cdot 10^{12}$	16			

Table S6. Summary of calibration curve and linearity range of catechins (n=6).

Analyte	logP	Retention time (min)	Regression equation	S_a	S_b	Linear range (μg/g)	Coefficient of determination r^2
**GC**	1.49	2.24	y=0.0062x + 0.0039	0.0001	0.0025	1 – 40	0.9988
**EGC**	1.49	2.83	y=0.0068x + 0.0025	0.0001	0.0028	1 – 40	0.9987
**C**	1.80	3.13	y=0.0111x + 0.0062	0.0002	0.0051	1 – 40	0.9983
**EC**	1.80	3.46	y=0.0168x – 0.0011	0.0003	0.0061	1 – 40	0.9990
**EGCG**	3.08	3.55	y=0.0283x + 0.0091	0.0005	0.0107	1 – 40	0.9989
**GCG**	3.08	3.68	y=0.0353x + 0.0068	0.0006	0.0128	1 – 40	0.9990
**ECG**	3.38	4.12	y=0.0434x+0.0053	0.0008	0.0171	1 – 40	0.9988

Table S7. Summary of accuracy, precision and recovery of catechins (n=6).

**Analyte**	**Concentration (μg/g)**	**Precision[a] (%RSD)**		**Accuracy[b] (%RE)**		**Recovery (%)**
		**Intra-day**	**Inter-day**	**Intra-day**	**Inter-day**	
**GC**	2.5	6.6	8.0	8.7	9.8	97.2
	20	3.2	4.6	1.4	3.3	98.5
	35	6.0	6.4	-4.7	-7.7	95.3
**EGC**	2.5	9.4	11	4.6	6.3	84.7
	20	2.6	3.5	-5.8	-8.6	97.2
	35	2.3	2.7	-7.6	-9.0	109
**C**	2.5	8.8	10	7.2	8.6	96.8
	20	3.7	5.7	-6.6	-7.2	86.5
	35	1.1	2.6	-2.3	-4.1	87.7
**EC**	2.5	7.6	8.5	4.5	5.6	80.2
	20	1.2	2.4	-4.4	-5.0	94.3
	35	1.8	1.7	-9.3	-10	85.2
**EGCG**	2.5	4.8	5.7	7.2	9.7	77.6
	20	2.6	5.0	-7.5	-9.3	85.2
	35	2.1	1.8	-8.9	-9.1	87.4
**GCG**	2.5	4.8	5.1	1.9	3.9	69.4
	20	2.4	3.7	-4.8	-5.6	74.1
	35	1.8	2.6	-7.6	-8.4	73.8
**ECG**	2.5	3.5	5.4	2.4	3.0	67.5
	20	1.7	4.0	-4.6	-6.0	76.3
	35	1.3	1.4	-9.3	-10	77.5

[a] Expressed as relative error (RE (%)).
[b] Expressed as relative standard deviation (RSD (%)).

Table S8. Content of catechins determined in infusion of tea.

Sample		Concentration (mg/g of dry weight[a])						
		GC	EGC	C	EC	EGCG	GCG	ECG
**Black tea**	**1**	8.37±0.04[b]	4.25±0.02	1.15±0.10	0.85±0.06	1.54±0.05	0.37±0.01	3.29±0.07
	**2**	12.4±0.30	2.73±0.08	0.98±0.02	0.38±0.03	2.52±0.01	0.19±0.01	3.30±0.04
	**3**	6.89±0.69	2.41±0.05	0.86±0.07	0.33±0.03	1.77±0.18	0.28±0.01	1.56±0.07
	**4**	14.1±1.27	4.21±0.53	0.54±0.04	0.64±0.03	3.40±0.83	0.34±0.03	3.29±0.40
	**5**	20.3±0.50	1.88±0.02	0.55±0.07	0.52±0.02	2.64±0.10	0.24±0.01	3.20±0.16
	**6**	2.47±0.04	2.19±0.01	1.09±0.02	0.92±0.09	2.14±0.07	0.06±0.01	1.29±0.01
	**7**	4.02±0.07	2.88±0.06	0.86±0.15	0.97±0.06	0.93±0.06	0.04±0.01	1.41±0.02
	**8**	8.23±0.07	0.24±0.02	0.19±0.07	0.34±0.01	0.33±0.08	0.11±0.01	0.59±0.01
	**9**	5.27±0.03	0.24±0.01	0.20±0.04	0.33±0.08	0.32±0.08	0.10±0.02	0.45±0.01
**Green tea**	**10**	4.10±0.29	14.9±0.85	0.16±0.04	3.23±0.18	14.5±1.54	2.61±0.20	3.02±0.30
	**11**	8.22±0.28	17.3±0.30	1.91±0.15	3.17±0.25	26.7±1.27	3.81±0.11	5.60±0.21
	**12**	16.6±0.90	15.8±0.21	1.23±0.14	2.34±0.30	31.2±1.06	0.80±0.09	8.47±0.35
	**13**	3.17±0.25	14.8±0.02	2.45±0.23	3.31±0.14	16.9±0.25	1.35±0.06	4.44±0.04
	**14**	7.88±0.14	12.2±0.48	1.75±0.23	2.78±0.12	20.3±0.12	2.65±0.06	3.79±0.04
	**15**	6.98±0.06	11.4±0.42	1.78±0.20	2.16±0.16	31.5±0.09	1.10±0.10	9.20±0.21
	**16**	5.37±0.15	7.66±0.09	1.99±0.16	5.40±0.15	23.7±0.12	3.41±0.03	8.88±0.01
	**17**	6.29±0.22	9.48±0.07	0.57±0.01	3.16±0.24	18.8±0.44	1.39±0.10	4.34±0.01
	**18**	7.49±0.16	16.3±0.74	0.20±0.02	3.90±0.13	24.8±0.37	3.96±0.08	9.79±0.03
**Fruit tea**	**19**	ND[c]	8.00±0.09	2.07±0.07	0.14±0.01	0.10±0.01	ND	0.11±0.01
	**20**	ND	6.00±0.08	1.11±0.06	0.12±0.01	0.13±0.01	ND	0.09±0.01

[a] Each value is the mean (μg/g of dry weight used for prepare of infusion) of three replications
[b] SD relative standard deviation
[c] ND not
